# Antibiotic Intervention Affects Maternal Immunity During Gestation in Mice

**DOI:** 10.3389/fimmu.2021.685742

**Published:** 2021-08-26

**Authors:** Marilen Benner, Alejandro Lopez-Rincon, Suzan Thijssen, Johan Garssen, Gerben Ferwerda, Irma Joosten, Renate G. van der Molen, Astrid Hogenkamp

**Affiliations:** ^1^Laboratory of Medical Immunology, Department of Laboratory Medicine, Radboud Institute for Molecular Life Sciences, Radboud University Medical Center, Nijmegen, Netherlands; ^2^Division of Pharmacology, Department of Pharmaceutical Sciences, Faculty of Science, Utrecht University, Utrecht, Netherlands; ^3^Department of Data Science, Julius Center for Health Sciences and Primary Care, University Medical Center Utrecht, Utrecht, Netherlands; ^4^Division of Immunology, Danone Nutricia Research B.V., Utrecht, Netherlands

**Keywords:** machine learning, placenta, mouse, gestation, pregnancy, antibiotics - immune effect, preeclampcia, offspring immunity

## Abstract

**Background:**

Pregnancy is a portentous stage in life, during which countless events are precisely orchestrated to ensure a healthy offspring. Maternal microbial communities are thought to have a profound impact on development. Although antibiotic drugs may interfere in these processes, they constitute the most frequently prescribed medication during pregnancy to prohibit detrimental consequences of infections. Gestational antibiotic intervention is linked to preeclampsia and negative effects on neonatal immunity. Even though perturbations in the immune system of the mother can affect reproductive health, the impact of microbial manipulation on maternal immunity is still unknown.

**Aim:**

To assess whether antibiotic treatment influences maternal immunity during pregnancy.

**Methods:**

Pregnant mice were treated with broad-spectrum antibiotics. The maternal gut microbiome was assessed. Numerous immune parameters throughout the maternal body, including placenta and amniotic fluid were investigated and a novel machine-learning ensemble strategy was used to identify immunological parameters that allow distinction between the control and antibiotic-treated group.

**Results:**

Antibiotic treatment reduced diversity of maternal microbiota, but litter sizes remained unaffected. Effects of antibiotic treatment on immunity reached as far as the placenta. Four immunological features were identified by recursive feature selection to contribute to the most robust classification (splenic T helper 17 cells and CD5^+^ B cells, CD4^+^ T cells in mesenteric lymph nodes and RORγT mRNA expression in placenta).

**Conclusion:**

In the present study, antibiotic treatment was able to affect the carefully coordinated immunity during pregnancy. These findings highlight the importance of inclusion of immunological parameters when studying the effects of medication used during gestation.

## Introduction

Any medication during pregnancy demands careful consideration. However treatment is essential when infections need to be controlled to ensure safe progression of the pregnancy. In western society, antibiotics for systemic use are amongst the most frequently prescribed drugs during gestation (1). Most commonly, gestational respiratory tract infections or urinary tract infections (UTI) require systemic anti-infectious medical intervention (2–4). If left untreated, 20-40% of asymptomatic UTI advance to acute UTI, which can lead to premature labor in up to half of the women affected (5). Antibiotic intervention has been shown to reduce complications of UTI such as preterm birth and/or low birth weight (6). Approximately 1 in 5 pregnancies is exposed to antimicrobial treatment nowadays (1, 7). While this can safeguard pregnancy, microbial intervention during pregnancy is known to have long-term effects on the offspring as well. Gestational use of antibiotics is associated with an increased risk for the offspring to develop non-communicable diseases like asthma, obesity, and even increased susceptibility to infections (8–10).

In general, antibiotic prescriptions require rational and critical use, not only to limit the selection towards drug-resistant pathogens (11). Awareness of the natural microbiomes’ contribution to physiology increases, and a diverse microbiome is key to healthy immunity (12–14). Systemic antibiotics drastically reduce the diversity of the gut microbiome and, depending on the compound and its target, impact on bacterial taxa can last for years (15, 16). This interference through broad-spectrum microbial modulation resulted, among others, in colonic infiltration of innate inflammatory cells in mice (17). In addition, antibiotic treatment skewed T cells towards an activated T helper (Th) 1 profile, together with a reduced proportion of FOXP3^+^CD4^+^ regulatory T cells (Treg) (17). After antibiotic treatment, a general increase in pro-inflammatory transcriptional and cellular responses was observed, such as an activation of dendritic cells and upregulation of genes of the pro-inflammatory cytokines interleukin 6 (IL-6), IL-8 and CXCL2 (18). The adverse effects of this inflammatory state might be limited in young adults (below 65 years of age) (18) but it is unsure if this holds true for pregnant women.

Pregnancy relies on tightly regulated immunity to allow trophoblast invasion and prevent infection, while excessive inflammation of the prenatal environment has to be avoided as well (19–27). Especially at the direct fetal environment, dysregulated immunity can cause pregnancy complications, such as preeclampsia (PE) and preterm birth, with long-term consequences on the offspring’s development (28–30). As such, antibiotic-mediated changes of maternal immunity can affect neonatal health. Although several studies have shown offspring immunity to be affected by prenatal microbial modulation (31–33), to our knowledge, the effects on maternal immunity have not been studied extensively.

Based on this, we questioned in this study whether gestational antibiotic use translates to maternal immune adaptations with possible impact on the feto-maternal interface. We here evaluated immunity of different maternal immunological compartments, including the placenta, using a murine model of gestational microbial modification.

## Materials and Methods

### Animals

8-week-old, specific pathogen-free, male C57BL/6 mice and 8-week-old nulliparous female BALB/c, purchased from Envigo (Horst, The Netherlands) were housed at the animal facility of the Utrecht University (Utrecht, The Netherlands) on a reversed 12 h light/dark cycle with unlimited access to water and semi‐purified AIN‐93G soy protein‐based rodent diet (Ssniff Spezialdiäten GmbH, Soest, Germany). Upon arrival, mice were habituated to the laboratory conditions for two weeks prior to the start of the study. The male mice were mated with a separate set of BALB/c females (Envigo, Horst, The Netherlands) prior to the experiment, and males with proven fertility were selected to mate with the experimental females. Males were housed individually before and after mating, and female mice were housed 2 per cage. Animal procedures were approved by the Ethical Committee for Animal Research of the Utrecht University and conducted according to the European Directive 2010/63/EU on the protection of animals used for scientific purposes (AVD108002016597).

### Experimental Design

**Figure 1:**
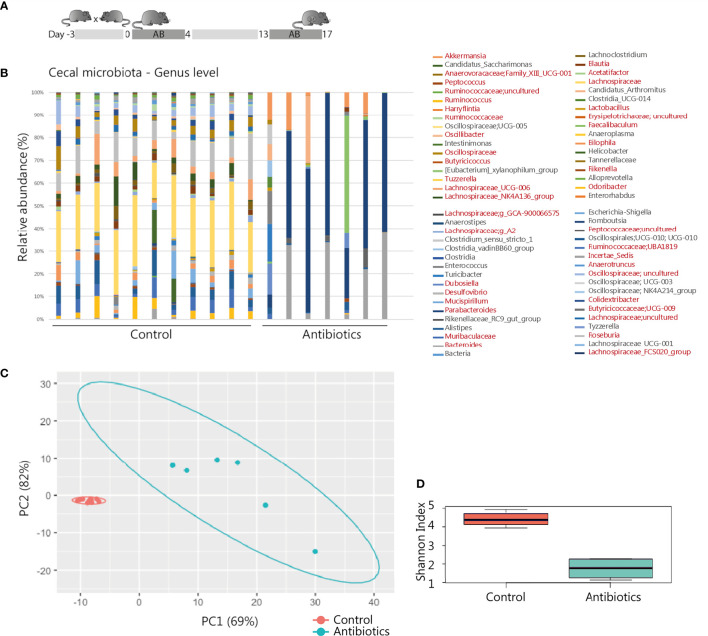
Antibiotic intervention in pregnant BALB/c mice. **(A)** Schematic representation of the experimental model. After randomly assigning mated mice to the treatment groups, 11 mice of the control and 8 mice of the treatment group were pregnant and included for further analysis. **(B)** 16S rRNA composition of the cecal samples per individual animal (1 sample of the antibiotic treatment group excluded due to read count <500). In the legend, genera shown in red were significantly affected by the treatment (see also **Supplementary Table T1**). **(C)** Principal component analysis of 16S rRNA sequencing data on genus level. **(D)** Shannon Index presenting microbial diversity.

After 14 days of acclimatization, bedding from the cages of assigned breeder males was added to the cages of experimental female mice to facilitate synchronization of the females’ cycle. After three days, males and females were housed together for 72 hours in a 1:2 ratio. Vaginal plugs were scored to assess the time of mating. After mating, the females were randomly assigned into the control group or the antibiotic treatment group. Antibiotic treatment was carried out by adding a mix of 2.5 mg/mL neomycin (Sigma Aldrich, Zwijndrecht, The Netherlands), 0.5 mg/mL metronidazole (Sigma Aldrich), and 0.09 mg/mL polymyxin (Sigma Aldrich) in the drinking water. The treatment consisted of two courses, starting with 4 days antibiotic treatment with followed by 10 days without antibiotics and another 4 days with antibiotics (**Figure 1A**). The animals were weighed before mating and at the end of experiment to evaluate the weight gain. The pregnant mice were killed by cervical dislocation on day 17 after mating, after which the number of fetuses and resorptions were assessed and tissue samples were collected for further analysis. During the sectioning, which was carried out in a laminar flowhood, sterile surgical instruments were used. Pregnant mice were killed in a separate part of the flowhood to avoid contamination of the location where murine tissues were collected. The skins of the mice were carefully swabbed with ethanol, after which an intraperitoneal lavage was carried out by flushing the peritoneal cavity with 2 ml of PBS to collect intraperitoneal leukocytes. Hereafter, the abdominal cavity was opened using one set of surgical instruments, carefully avoiding contact of the skin with the abdominal cavity by pinning the skin of the animals back. Another set of sterilized surgical instruments was used to isolate placental and fetal tissues, and amniotic fluid was collected from individual amniotic cavities of the fetuses. Hereafter, other tissues (e.g. spleen, intestinal tissue and lymph nodes) were isolated, for which purpose the carcass of the mouse was moved to another section of the laboratory, to avoid contamination of the laminar flowhood. Samples isolated for analysis of mRNA-expression were immediately snap-frozen using dry ice and stored at -80°C. Samples used for flow cytometry or cell culturing were kept on ice until further processing.

### Microbiota-Analysis Placenta & Cecum

Total DNA was isolated from 50-225 microgram of cecal content feces and 125-200 microgram of placental tissue using the QIAamp Stool DNA mini kit (Qiagen). DNA was quantified by NanoDrop assay. The 16S rRNA gene profiling was analyzed as described by Paganelli et al., 2019, by the Exposome HUB Utrecht. Briefly, 16S rRNA regions V3 and V4 were sequenced with an Illumina MiSeq reagent Kit v3 (600‐cycle) on an Illumina MiSeq instrument (Illumina) (34). Samples were analyzed with the QIIME™ 2 microbial community analysis pipeline (35). For the cecum samples, significant differences between treatment and control groups at genus level were detected using the statistical framework analysis of composition of microbiomes (ANCOM) (36). p‐values were adjusted for multiple comparisons using false discovery rates. RStudio 1.4.1103 (RStudio Team) was used to calculate alpha diversity using the Shannon index, and significance was calculated by the Wilcoxon test. The global difference in microbiota composition was assessed using principal component analysis (PCA), employing zCompositions, centered log‐ratio (CLR) transformation, and ggplot R packages.

### SCFA Analysis Cecal Content

The cecal SCFA levels of acetic, propionic, butyric, isobutyric and valeric acids were quantitatively determined as well as levels of lactic acids as described previously (37, 38). The SCFA were captured using a Shimadzu GC2010 gas chromatograph (Shimadzu Corporation, Kyoto, Japan) equipped with a flame ionization detector. SCFA concentrations were determined using 2-ethylbutyric acid as an internal standard. Lactic acids were determined enzymatically using a d/l-lactic acid detection kit with d- and l-lactate dehydrogenase (EnzyPlus, BioControl Systems, Inc., Bellevue, WA, USA).

### Lymphocyte Subset Analysis

For flow cytometric analysis of lymphocytes, single cells suspensions were prepared from intraperitoneal lavage, isolated placental tissue, spleens, mesenteric lymph nodes (MLN) and inguinal lymph nodes (ILN). ILN and MLN single cell suspensions were obtained by crushing the tissue through 70 µm cell strainers on ice. The strainers were washed with RPMI 1640 medium, after which the cells were counted, resuspended in PBS and kept on ice until further processing. Splenocytes were similarly isolated, but red blood cells were lysed prior to counting the cells using lysis buffer (8.3 g NH4Cl, 1 g KHCO3, and 37.2 mg EDTA dissolved in 1 L demi water and filter sterilized). Placental tissues were cut into small pieces and incubated with Accutase (Stempro, GIBCO Life Technologies, Waltham, USA) for 35 min at 37°C under slight agitation. Hereafter, red blood cells were lysed as described for splenocytes, and placental cells were washed, counted, resuspended in PBS and kept on ice until further processing. Prior to staining cells for flow cytometric analysis, they were washed in PBS and 50µl of cell suspension (4.10^6^ cells/mL) was incubated with a fixable viability dye eFluor^®^ 780 (eBiosciences, Thermo Fisher Scientific, San Diego, CA, USA) for 30 min at 4°C. After washing, cells were incubated with anti-mouse CD16/CD32 (1:100 dilution in PBS/1% BSA; Mouse BD Fc Block, BD Pharmingen, San Jose, CA, USA) to block non-specific binding sites. For flow cytometric analysis of surface marker expression, cells were incubated at room temperature for 1 h in the dark with corresponding antibody-cocktails, washed with PBS/1% BSA and fixed in 1% paraformaldehyde-solution until flow cytometric analysis. For the analysis of intracellular markers, cells were first stained for extracellular markers, washed with PBS/1% BSA and incubated overnight in Fix/Perm buffer (eBiosciences). The following day, cells were washed with permeabilization buffer (eBioscience), and incubated with anti-mouse CD16/CD32 for 15 min at 4°C in the dark. Next, the cells were stained for intracellular markers for 30 min at 4°C in the dark, washed in PBS/1% BSA and immediately used for flow cytometric analysis. The following fluorochrome-conjugated monoclonal antibodies were used: CD4-PerCP-Cy5.5 (eBioscience), CD69-APC (eBioscience), CXCR3-PE (eBioscience), T1ST2-FITC (MD Biosciences, St. Paul, MN, USA); CD11b- PerCP-Cy5.5 (eBioscience), NK1.1-APC (eBioscience), CD49b-FITC (eBioscience), CD94-PE (eBioscience); CD4- Brilliant Violet 510, CCR6-PE (BioLegend, San Diego, CA, United States), CD25-PerCP-Cy5.5, (eBiosciences), CD196 (CCR6)-PE (BioLegend), CD127-PE-Vio770 REA (Miltenyi Biotec, Bergisch Gladbach, Germany), Neuropilin-eFluor450 (eBioscience), RorγT-Alexafluor 647 (BD Pharmingen, San Jose, CA, USA), CD1d-PerCP-Cy5.5 (BioLegend), CD5-Alexa Fluow 647 (BioLegend), CD19-PE-Cy7 (BD), CD21/CD35-FITC (BD), CD23-PE (BD), CD24-Brilliant Violet 510 (BD), Tim-1-Brilliant Violet 421 (BD), Viability-APC-Cy7 (eBioscience). Results were collected with BD FACSCanto II flow cytometer (Becton Dickinson, Franklin Lakes, NJ, USA) and analyzed with FlowLogic software (Inivai Technologies, Mentone, VIC, Australia) and Kaluza software (v2.1, Beckman Coulter, Fullerton, CA, USA).

### Placental and Intestinal mRNA-Expression Analysis

Total RNA was isolated from maternal intestinal tissues and placenta using the RNeasy mini kit (Qiagen, Germantown, USA) and cDNA was prepared using the iScript cDNA synthesis kit (Bio Rad, Veenendaal, the Netherlands), according to the manufacturer’s instructions. For quantitative real-time PCR, the reaction mixture was prepared by adding specific forward and reverse primers and iQSYBR Green Supermix (Bio-Rad Laboratories, Hercules, CA, USA) to the cDNA samples, and amplifications were performed according to the manufacturer’s instructions using the CFX96 Touch™ Real-Time PCR Detection System (Bio-Rad Laboratories, Hercules, CA, USA). Validated qPCR primers for FOXP3, T-bet, GATA3, ROR-γT, β-Actin and IL-10 were obtained from SABiosciences (Qiagen, Germantown, USA). mRNA expression levels were calculated relative to the expression of β-actin reference gene with CFX Manager software (version 1.6).

### Determination of Cytokine Profiles (in Amniotic Fluid, and After *Ex Vivo* Stimulation of Splenocytes)

Splenocytes collected from pregnant mice were cultured at a concentration of 4.10^6^ cells/mL RPMI 1640 culture medium in 96-well U-bottom culture plates at 37°C in a humidified environment containing 5% CO_2_, in the presence or absence of 10 µg/mL lipopolysaccharide (LPS) (Sigma). Cell culture supernatants were collected after 24 hours and stored at −20°C until further analysis. A ProcartaPlex multiplex protein assay kit (Invitrogen, Thermo Fisher Scientific, Waltham, MA, USA) was used to assess the concentrations of interleukin (IL)-1β, IL-2, IL-4, IL-6, IL-10, IL-22, tumor necrosis factor (TNF)-α, and interferon (IFN)-γ in amniotic fluid and cell culture supernatants, according to manufacturer’s instructions. To calculate expression levels of cytokines by maternal splenocytes, cytokine concentrations in supernatants of LPS-stimulated cells were corrected for those measured in supernatants of unstimulated cells.

### Recursive Automatic Ensemble Feature Selection

To discover the selection of immunological parameters that allow classification as control or antibiotic-treated group, a previously established ensemble feature selection was used (39, 40). This strategy allows for a more general selection of stratifying features than a single classifier, overcoming the bias of each individual algorithm. In brief, 8 classifiers (Bagging, Gradient Boosting, Logistic regression, Passive-Aggressive regression, Random Forest, Ridge Regression, SGD (Stochastic Gradient Descent on linear models), and SVC (Support Vector Machines Classifier with a linear kernel) classifier) generated a list of relative feature importance that is scored for a combined summary of top most relevant features. To ensure generality of the results, each classifier was run 10 times together with a 10-fold cross validation. This was repeated in a stepwise reduction of the 129 initial features by 20%, while determining the accuracy for each classifier.

### Univariate Analysis

Data were analyzed using R v.4.0.2 and the ggpubr, ggplot2, ggsignif, tidyr packages. Non-parametric Mann-Whitney test was performed. Values of p < 0.05 were considered statistically significant.

## Results

### Microbial Disruption Does Not Result in Pregnancy Complications in Mice

Gestational antibiotic intervention of metronidazole, neomycin and polymyxin was previously shown to influence immunity of the offspring without observed pregnancy complications (41). Following this established treatment regime (**Figure 1A**), we assessed effective maternal microbial modification. 16S rRNA analysis of the gut showed a distinct microbiota composition in antibiotic-treated mice compared to the control group (**Figures 1B, C**). In total 39 different genera showed a significant change in relative abundance as well when both groups were compared using ANCOM pipeline (**Supplementary Table S1**). In addition, a significant decrease of total alpha diversity represented by Shannon index diversity was observed in the antibiotic-treated group (p-value = 0.0001616, **Figure 1D**). As major microbiota metabolites, SCFAs are involved in regulating intestinal integrity and intestinal immunity (42). Antibiotic treatment did not affect SCFA levels in maternal cecum, as no significant differences were observed regarding levels of acetic acid, propionic acid and butyric acid (**Supplementary Figure S1A**). Iso-butyric acid, valeric acid, and iso-valeric acid were not quantifiable in the cecal content of all sampled mice. To investigate a possible direct microbial effect of treatment on the prenatal environment, the 16S rRNA profiles of placental tissue were analyzed as well. Although some samples had reads as revealed by Qiime2 and DADA2 analysis, these represented mostly unspecific contamination, probably due to the high concentration of eukaryotic DNA in the samples. Therefore, based on our methods, we could not identify the presence of a specific bacterial community in the placentas analyzed. Pregnancy outcome was assessed as the number of pregnancies, intact and resorbed fetuses. No statistically significant differences were observed between groups. Eleven pregnant mice of the control group, and 8 of the antibiotic-treated group had a mean litter size of 7.8 (range 3-11, mean resorption rate 1) and 9.4 (range 3-11, mean resorption rate 0.8), respectively (**Supplementary Figures S1B, C**). No clinical symptoms in the antibiotic-treated group were observed that would suggest adverse effects on maternal health and, as a consequence, pregnancy. Building on this intervention model, we then proceeded to analyze the consequences of antibiotic treatment on the maternal immune system.

### Antibiotic Treatment Associated With Shift in Immune Parameters Differentiating From Control Group

**Figure 2:**
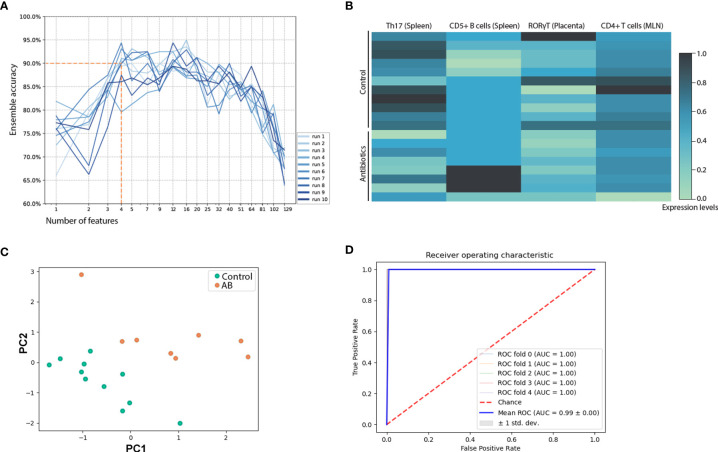
Multivariate analysis of immunologic assessment across samples of spleen, mesenteric lymph node, inguinal lymph node, peritoneal cavity lavage fluid, amniotic fluid, and placenta. **(A)** Recursive feature reduction used in an ensemble machine-learning strategy to determine the number of top features needed to achieve robust (>90% accuracy) classification. **(B)** Top 4 immune features that allow for distinction between s. Normalized expression levels are depicted. **(C)** Principal component analysis based on the top 4 features that allowed for optimal classification. **(D)** Individual classification algorithms were run with the top 4 features of the ensemble ranking. The receiver operating curve of Ridge regression is shown. The same results were observed using Passive-Aggressive or Logistic regression. Additional receiver operating curves are depicted in **Supplementary Figure S2**. AB, treated with antibiotics; MLN, mesenteric lymph nodes.

Maternal immunity was assessed by flow cytometry of placenta, spleen, ILN, MLN and peritoneal cavity lavage fluid (PCLF), mRNA of intestines and placenta, and cytokine levels of amniotic fluid and supernatants of *ex vivo* splenocyte-cultures. A total of 129 different parameters were analyzed (**Supplementary Table S2**). To detect the classifying immune alterations occurring upon antibiotic treatment, a previously validated machine-learning ensemble classification strategy was used (39, 40), which is suited for robust feature selection in the given low sample size setting. This method combines 8 classification algorithms, which compensates for possible biases inherent to the individual algorithm. The output of the 8 classification algorithms each yielded a ranked list of features, representing the contribution of the 129 immune features to classification. Each list was weighted based on coefficients and frequency of an individual feature contributing to classification (40) to aggregate classifiers to a single ranking. Classification algorithms were repeatedly run using the top 80% of the ensemble ranking (recursive feature selection). Each classification run was carried out 10 times, each run being subjected to 10-fold cross validation. At a global average accuracy of 90% as cutoff to ensure robust classification (**Figure 2A**), ensemble accuracy for each iteration of feature combinations showed optimal classification when combining 4 features: frequency of Th17 cells and CD5^+^ B cells of the spleen, fold-change of RORγT assessed in placental tissue, and frequencies of CD4^+^ T cells of MLN (normalized expression values, **Figure 2B**). Principal component analysis illustrated separation of maternal immunity after antimicrobial intervention from healthy control (**Figure 2C**). The area under the curve (AUC) of 0.99 of the receiver operating characteristic (ROC) analysis confirmed robust classification based on the 4 identified features (**Figure 2D**).

### Systemic and Placental T Cell Adaptations Mediated by Gestational Antibiotics

**Figure 3:**
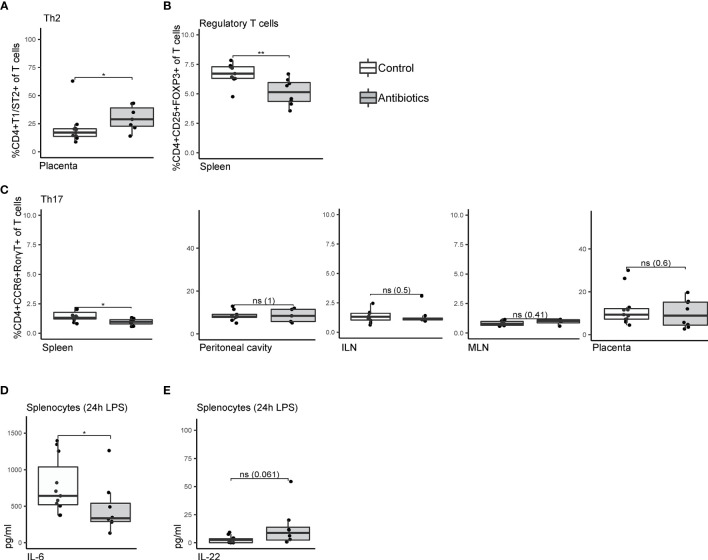
Univariate analysis comparing T cell subsets of antibiotic-treated and control mice. **(A)** Frequencies of T helper 2 cells within T cells isolated from placental tissue, staining CD4^+^T1ST2^+^. **(B)** Splenic CD4^+^CD25^+^FOXP3^+^ regulatory T cells as frequency of total T cells. **(C)** CD4^+^CCR6^+^RORT^+^ T helper 17 cells isolated from spleen, peritoneal cavity lavage fluid, inguinal lymph nodes, mesenteric lymph nodes, and placenta. **(D)** Concentrations of IL-6 and **(E)** IL-22 in supernatant of splenocytes after 24h lipopolysaccharide stimulation. Cytokine expression levels were corrected for concentrations measured in unstimulated splenocytes. Data were compared by Mann-Whitney/Wilcoxon tests (non-parametric), *p < 0.05 **p < 0.01. ILN, Inguinal lymph nodes; LPS, lipopolysaccharide; MLN, mesenteric lymph nodes; ns, non-significant.

Immune features of adaptive immunity contributed to stratification as shown by machine-learning. Additionally, by univariate analysis, extra attention was payed to the different T cell subsets, whose differentiation is known to be influenced by symbiotic microbiota (43–45). While frequencies of Th1 (CD4^+^CXCR3^+^) and Th2 (CD4^+^T1/ST2^+^) (46) in spleen, ILN, MLN and PCLF were not affected by treatment (**Supplementary Figures S3A, B**), a significant increase in placental Th2 cell frequencies was observed (29.0% ± 2.8% compared to 20.8% ± 4.4% in the control group, **Figure 3A**). Percentages of CD4^+^CCR6^+^RorγT^+^ Th17 cells were lower in the spleens of mice treated with antibiotics (1.4% ± 0.1% compared to 1.1% ± 0.1% in the control group, **Figure 3B**) but no such differences were observed in other tissues. A significantly lower percentage of splenic CD4^+^CD25^+^FOXP3^+^ regulatory T cells (Treg) was observed in the antibiotic-treated group (5.3% ± 0.4% compared to 6.7% ± 0.3% in the control group, **Figure 3C**), but not in other compartments (**Supplementary Figure S3C**). Overall, T cell activation as observed through CD25 and CD69 expression was not affected in any of the tissues (**Supplementary Figure S3D**). Functional assessment of maternal lymphocytes through splenocytes stimulated *ex vivo* with LPS revealed only limited alterations of detectable cytokine levels in the supernatant. IL-6 production was significantly affected by the antibiotic treatment (**Figure 3D**) and a trend towards a higher IL-22 production was observed (p 0.061, **Figure 3E**). No significant differences were observed for any of the other cytokines analyzed (IFNγ, TNFα, IL-1β, IL-10, IL-2).

## Discussion

In utero exposure to antibiotics could impact neonatal immunity through two different routes: by disrupting microbial colonization of the child, needed for healthy immune development, or by the antibiotics’ impact on maternal immunity. The latter is associated with pregnancy complications known to alter offspring development (47–50). In the current study, we examined the effect of antibiotic treatment on maternal immunity using a murine gestational model. The chosen microbial intervention strategy was shown to affect offspring immunity (41). In line with this previous investigation of this combination of antibiotics, antibiotic treatment significantly decreased microbial diversity in the ceca of pregnant mice, but none of the mice were observed to have any clinical symptoms or reduced reproductive success. It was however possible to detect a maternal shift in immunological profile by using a machine learning ensemble classification strategy to assess >100 immune parameters of different sampling sides simultaneously (39, 40). Recursive feature elimination reduced the assessed parameters to a selection of 4 immunological features that distinguished control from antibiotic-treated mother animals with an accuracy of >90%. Of note, immune adaptations were observed throughout the maternal body, reaching as far as the placenta.

Antibiotics are known to affect intestinal immunity due to microbial manipulation of this immunological compartment (17, 51). Little is known on how this translates to other immune sites, especially regarding gestational tissues. In this exploratory approach, samples of placenta and amniotic fluid were included, and immune features were obtained through multiple methods, i.e. a combination of flow cytometry, mRNA expression, and analysis of soluble factors, to cover a wide range of possible effects. Using machine-learning and recursive feature selection allowed for an open assessment of studied parameters. This dimensionality reduction enabled an unbiased focus on the strongest induced changes. Additionally, the applied ensemble strategy offered stable feature selection in this low sample size setting. After mating, of the 40 females that were randomly allocated to the control and treatment group, 11 and 8 mice respectively were pregnant and available for a final readout. Small sample size and large variation of the input data could weaken reproducibility when single selection algorithms are used, a limitation that could be overcome by the applied ensemble approach (40, 52). The presented high accuracy of the classification underlines how computational methods may help to reduce the number of animals needed for *in vivo* studies.

While earlier findings showed that antibiotic treatment resulted in a macrophage-dependent increase in inflammatory colonic Th1 responses mice (17), we did not observe any differences in Th lymphocyte populations in the MLN, the gut-draining lymph nodes, of the antibiotic-treated animals. Of note, the study by Scott and colleagues investigated immunity in male mice (17) and thus could not take into account the highly specialized immune dynamics of pregnancy. We additionally investigated placental and amniotic fluid samples, showing that systemic immune features do not represent immunity of gestational tissues. For example, placental CD4^+^CD25^+^FOXP3^+^ Treg populations remained unaffected, whereas the percentage of splenic Treg in antibiotic-treated mice was reduced. Especially Treg of the fetal-maternal interface are considered critical to maintaining the anti-inflammatory environment necessary during the implantation period and throughout gestation (53). The increase in decidual Treg upon conception is hypothesized to be facilitated locally; through seminal fluid (54), human chorionic gonadrotropin secreted by the blastocyst (55), extravillous trophoblast cells (56), or local immune cells such as decidual macrophages (56). This local induction, independent from systemic immunity, might be connected to the observed lack of antibiotic effect on placental Treg. On the other hand, we detected an increase in the Th2 cell populations in the placentas after antibiotic treatment. Based on the premise that gestational immunity depends on a tightly regulated Th1/Th2 mediated cytokine balance (57), it is tempting to consider this a protective counteraction to prevent from a possible proinflammatory load upon external modifications during gestation. Thus, while the underlying mechanisms of how gestational antibiotics may perturb maternal immunity and fetal development are not yet clear, our results emphasize the need to study parameters of the fetal environment. The observed altered immunological profile upon antibiotics may be linked to side effects that were previously exclusively ascribed to its antimicrobial effect.

During pregnancy, any dysregulation of immunity might affect placentation and thus fetal development (21, 58–60). Gestational immune adaptations are highly specialized to enable selective tolerance towards invading fetal cells, immune-competence to overcome pathogenic invasion, and immune-mediated support of establishing vascularization during placenta formation (61–64). Imbalance of immunity is thought to hamper correct placentation and thus contribute to the etiology of preeclampsia (PE) (65, 66). Indeed, prescription of antibiotics during pregnancy is associated with an increased risk of PE, which is also concerning considering that a fairly large proportion of pregnant women are prescribed antibiotics without an indication (67, 68). Still, it is unclear whether infections like UTI themselves, or their treatment is associated with an increased risk of developing PE (69). In case of an infection that requires antibiotic intervention, the involved inflammatory cascade may elicit systemic maternal inflammation and endothelial injury, which could also increase the risk of PE (70–73). Nevertheless, *in vitro* assays have shown that an alteration of immune responses can also occur independent of altered microbiota as phagocytosis by macrophages was inhibited directly through addition of antibiotics (74). Moreover, the current study shows that also in absence of infection, antibiotics affect the immune balance during pregnancy. While the results from the current study cannot directly link antibiotics use to the development of PE, we show that also that also in the absence of infection, antibiotics affect immune balance during pregnancy. This dysregulated immune response, reaching as far as the placenta, could impact upon the progression of implantation and placentation.

In conclusion, either mediated by manipulation of the microbial profile or by direct effects of antibiotics, treatment affects the tightly regulated immunity of pregnancy. The associated poorly understood - but possibly far-reaching - consequences underscore the need for careful assessment and restraint use of antibiotics during pregnancy. Pregnant women are generally excluded from clinical trials, and, other than what can be deduced from retrospective studies, few approaches take into account the unique adaptations occurring to maintain a healthy pregnancy. These present results highlight the importance of *in vivo* studies on medication used during gestation to employ pregnancy models, taking into account the unique immunological properties, and possible tissue-specific effects, of this period.

## Data Availability Statement

The original contributions presented in the study are included in the article/**Supplementary Material**. Further inquiries can be directed to the corresponding author. Sequencing data are accessible at the European Nucleotide Archive, accession number PRJEB46451.

## Ethics Statement

The animal study was reviewed and approved by Ethical Committee for Animal Research of the Utrecht University.

## Author Contributions

Conceptualization: MB, AH, RM, JG, IJ, and GF. Methodology: MB, AH, and ST Investigation: MB, AH, ST, and AL-R. Writing: MB and AH. All authors contributed to the article and approved the submitted version.

## Funding

MB is supported by a junior researcher grant of the Radboud Institute for Molecular Life Sciences.

## Conflict of Interest

JG is a Director of Immunology at Nutricia Research, Netherlands.

The remaining authors declare that the research was conducted in the absence of any commercial or financial relationships that could be construed as a potential conflict of interest.

## Publisher’s Note

All claims expressed in this article are solely those of the authors and do not necessarily represent those of their affiliated organizations, or those of the publisher, the editors and the reviewers. Any product that may be evaluated in this article, or claim that may be made by its manufacturer, is not guaranteed or endorsed by the publisher.
