# Supplementary material for: Antibiotic Intervention Affects Maternal Immunity During Gestation in Mice
Source: Front Immunol. 2021 Aug 26;12:685742. doi: 10.3389/fimmu.2021.685742 (PMC8428513; doi:10.3389/fimmu.2021.685742)
Supplement: Supplementary file 1 [file DataSheet_1.pdf]

Supplementary Material

Antibiotic intervention affects maternal immunity during gestation in mice

Marilen Benner, Alejandro Lopez-Rincon , Suzan Thijssen, Johan Garssen, Gerben Ferwerda, Irma Joosten, Renate G. van der Molen, Astrid Hogenkamp

Supplementary Figure S1-4
Supplementary Table T1, T2

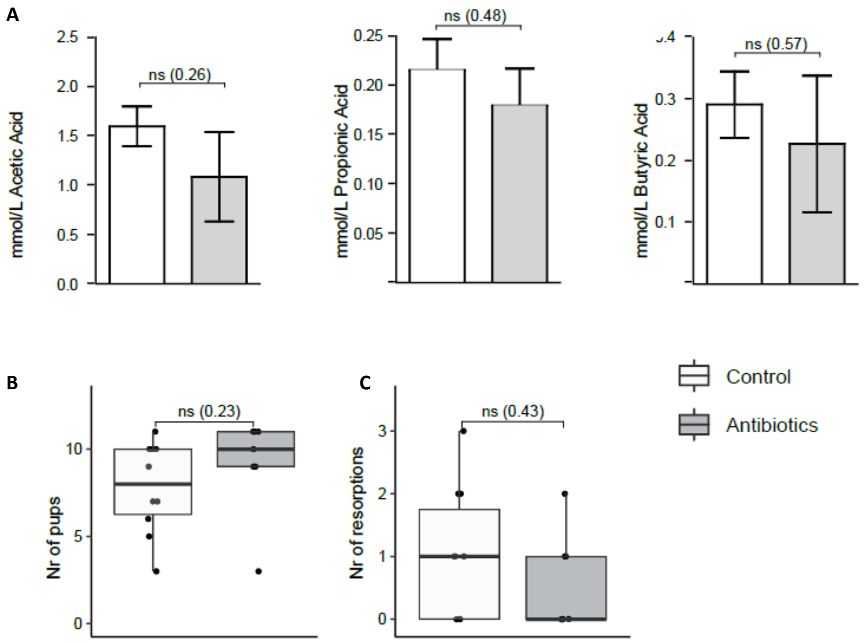

Supplementary Figure S1 | (A) Short chain fatty acids of maternal cecum. **(B)** Reproductive outcome assessed as litter size, and (**C)** numer of resorptions per animal. Data were compared by Mann-Whitney/Wilcoxon tests (non-parametric), ns: non-significant.

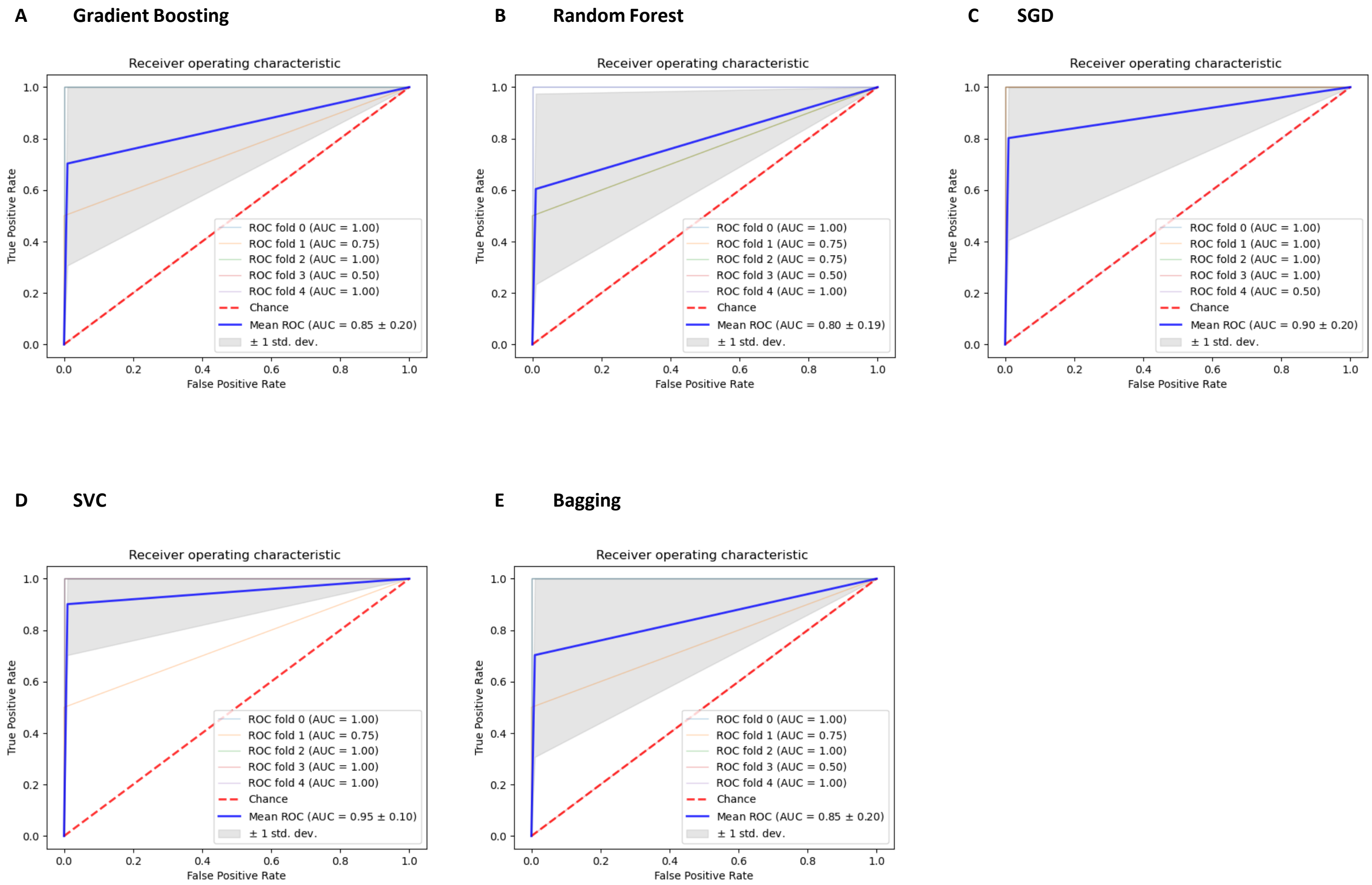

Supplementary Figure S2 | Receiver operating characteristic curves of the individual classifiers used in the ensemble strategy after feature selection.

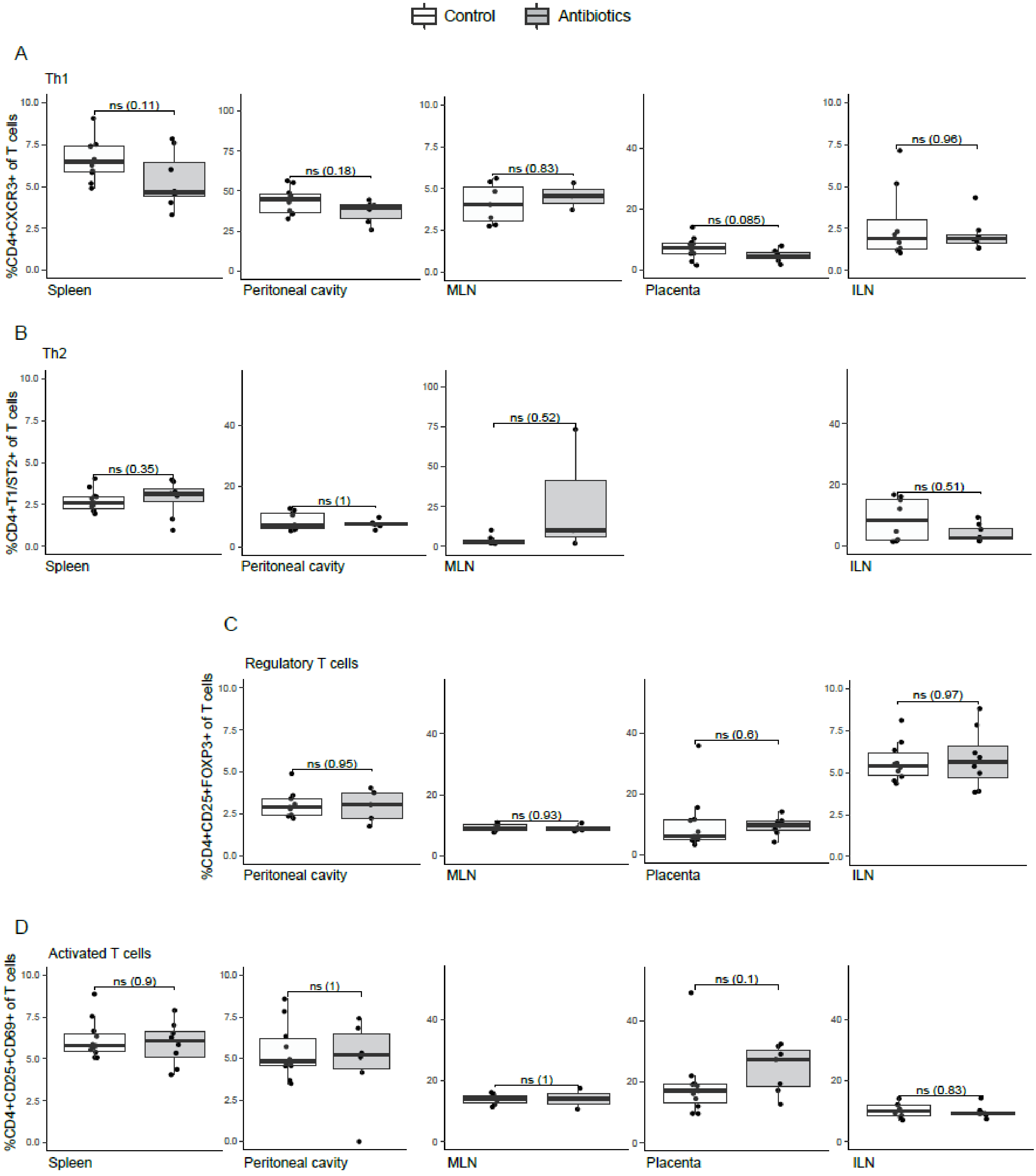

Supplementary Figure S3 | Univariate analysis of T cells. **(A)** T helper 1 cells, **(B)** T helper 2 cells, **(C)** Regulatory T cells, and **(D)** activated T cells. ILN: inguinal lymph nodes, MLN: mesenteric lymph nodes, ns: non-significant, Th: T helper cells.

A

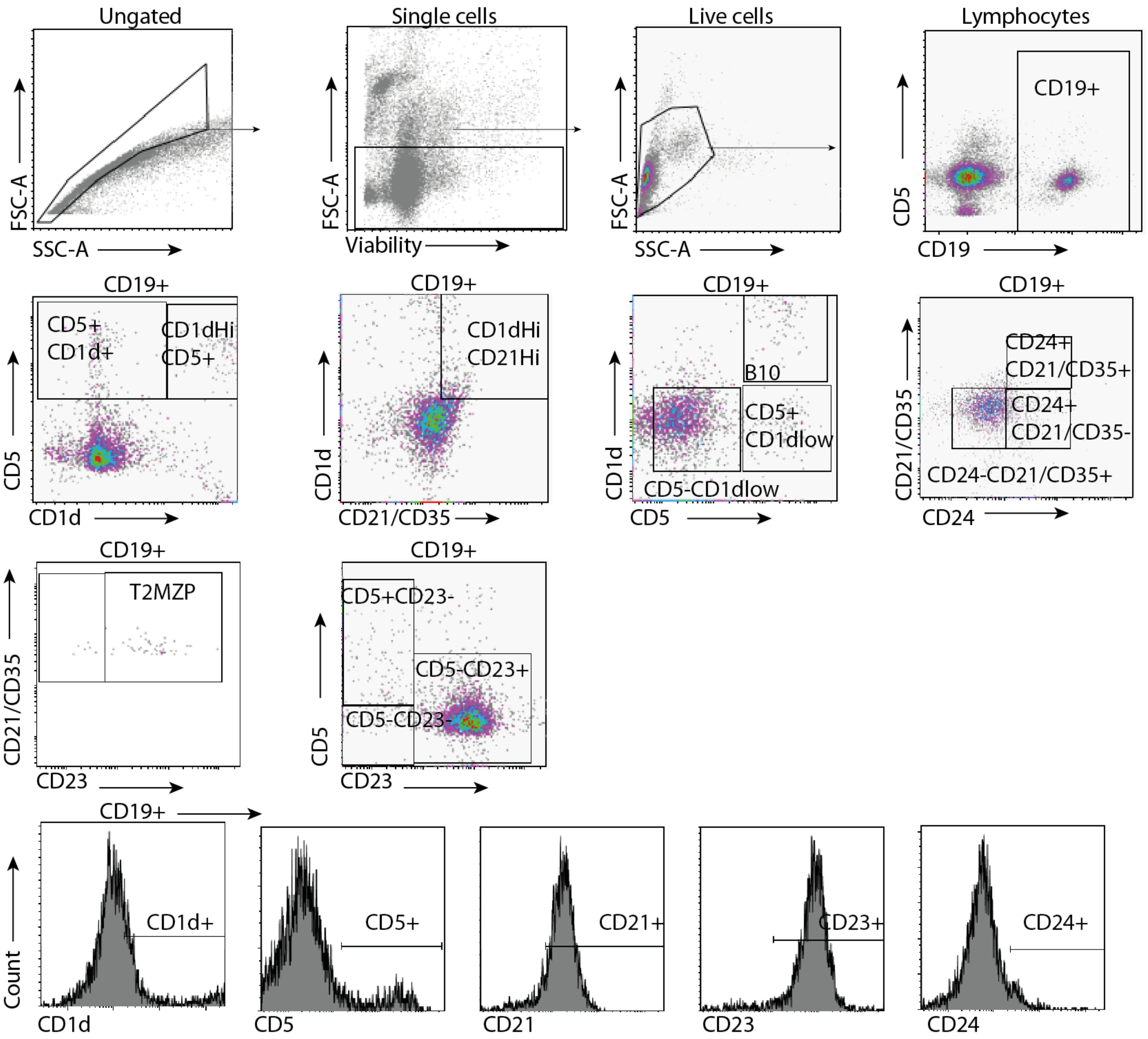

B

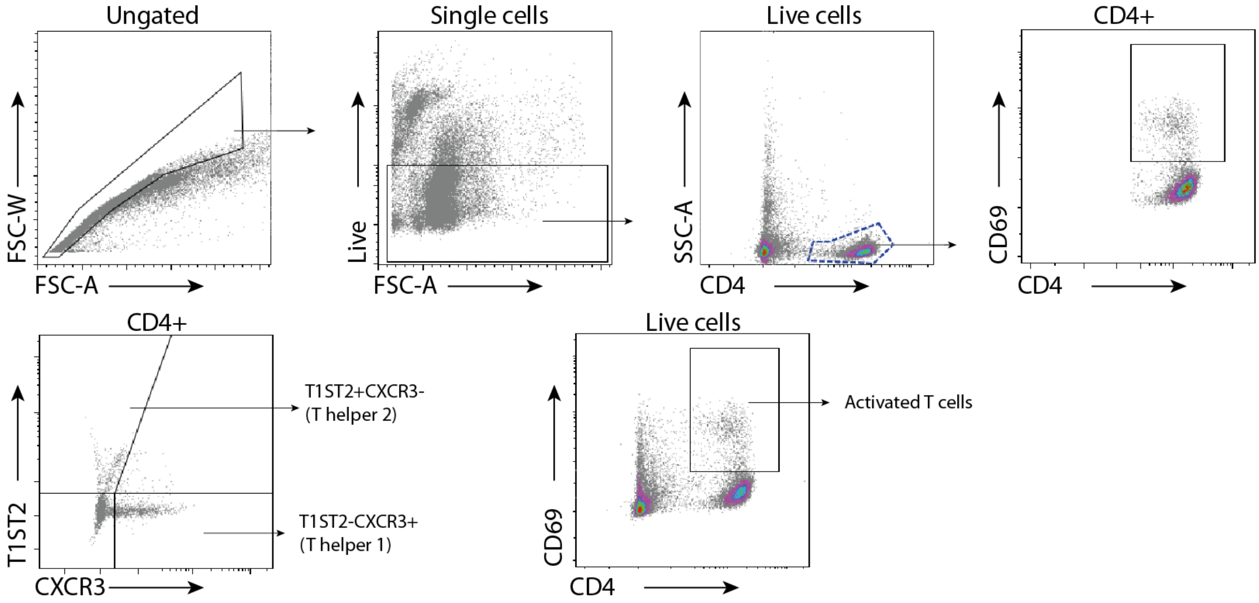

Supplemental Figure S4 | Gating strategy of features included in machine learning approach (see Supplemental Table T2 for list of features), continued on page 5.

C

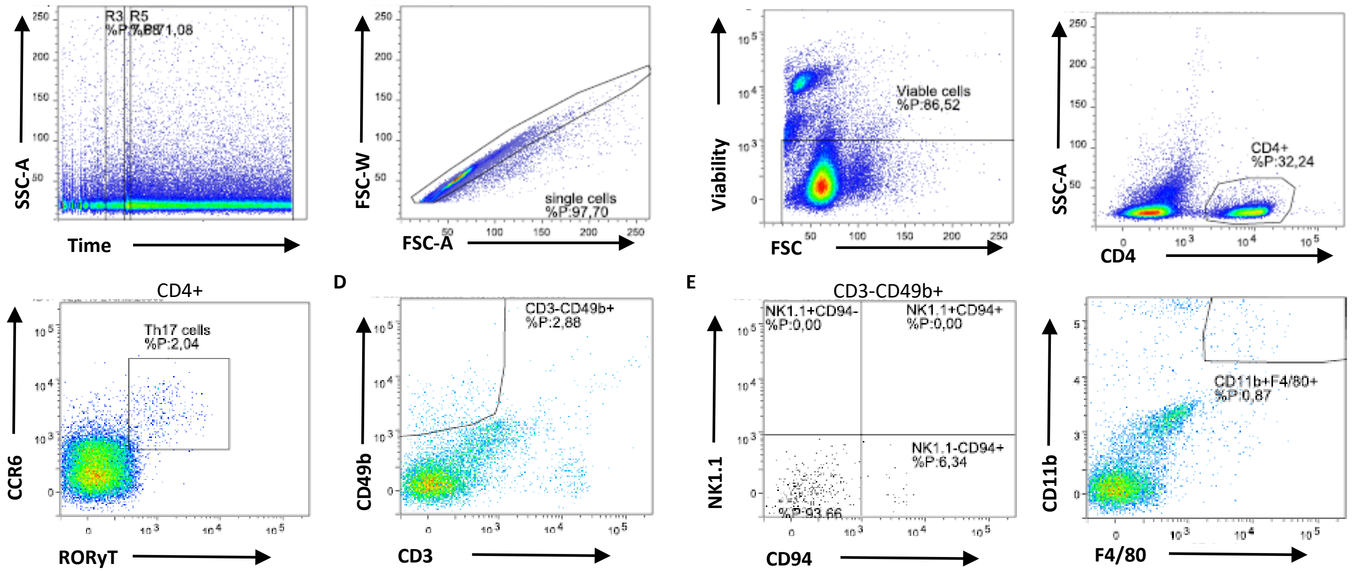

Supplementary Figure S4 continued | Gating strategy of assessed immune features (see Supplemental Table T2 for list of features). **(A)** Flow cytometry plots of B cells and **(B-C)** T cells from spleen, **(D)** – **(F)** flow cytometry plots of macrophages and NK cell features from placenta. Gates were set based on fluorescence minus one stainings and isotype controls to adjust for autofluorescence. Single stainings were performed to calculate compensation.

Supplementary Table T1 | Genera affected by antibiotic treatment as observed through 16S rRNA analysis of cecal microbiota.

**Microbiota (Family, genus)**	**Average**	**SD**	**Average**	**SD**
Bacteroidaceae_Bacteroides	0.006759	0.013215	0.187006	0.1681278
Marinifilaceae, g.Odoribacter	0.039366	0.031529	0.000874	0.0023117
Muribaculaceae, g.Muribaculaceae	0.047996	0.027715	0.005422	0.009187
Rikenellaceae, g.Rikenella	0.009005	0.007054	0.002075	0.0054903
Tannerellaceae, g.Parabacteroides	0.002205	0.003033	0.456937	0.2284479
Deferribacteraceae, g.Mucispirillum	0.041651	0.039894	0	0
Desulfovibrionaceae, g.Bilophila	0.025431	0.01803	0	0
Desulfovibrionaceae, g.Desulfovibrio	0.013822	0.008256	0	0
Erysipelotrichaceae, g.Dubosiella	1.88E-05	6.23E-05	0.0303	0.0519778
Erysipelotrichaceae, g.Faecalibaculum	3.75E-05	0.000125	0.079191	0.1937113
Erysipelotrichaceae, g.uncultured	0	0	0.0011	0.0020637
Lactobacillaceae, g.Lactobacillus	0.007371	0.006079	0.002768	0.005115
Lachnospiraceae	0.25393	0.047479	0	0
Lachnospiraceae, g.A2	0.01104	0.006533	0	0
Lachnospiraceae, g.Acetatifactor	0.004405	0.004506	0	0
Lachnospiraceae, g.Blautia	0.020167	0.010525	0.00273	0.0072241
Lachnospiraceae, g.GCA900066575	0.005693	0.004069	0	0
Lachnospiraceae, g.Lachnoclostridium	0.021349	0.016686	0	0
Lachnospiraceae, g.Lachnospiraceae_NK4A136_group	0.040543	0.03569	0.000135	0.0003574
Lachnospiraceae, g.Lachnospiraceae_UCG006	0.048969	0.03913	0	0
Lachnospiraceae, g.Roseburia	0.100611	0.045594	0	0
Lachnospiraceae, g.Tuzzerella	0.004984	0.002313	0	0
Lachnospiraceae, g.uncultured	0.015858	0.009272	0	0
Butyricicoccaceae, g.Butyricicoccus	0.000863	0.000516	0	0
Butyricicoccaceae, g.UCG009	0.001958	0.001477	0	0
Oscillospiraceae	0.042516	0.023665	0.002801	0.0074111
Oscillospiraceae, g.Colidextribacter	0.024567	0.017683	0	0
Oscillospiraceae, g.Oscillibacter	0.00919	0.007008	0	0
Oscillospiraceae, g.uncultured	0.039401	0.02487	0	0
Ruminococcaceae	0.009283	0.006574	0	0
Ruminococcaceae, g.Anaerotruncus	0.007639	0.005925	0	0
Ruminococcaceae, g.Harryflintia	0.000823	0.001249	0	0
Ruminococcaceae, g.Incertae_Sedis	0.014978	0.006661	0	0
Ruminococcaceae, g.UBA1819	0.000903	0.000805	0	0
Ruminococcaceae, g.uncultured	0.012825	0.005037	0	0
Peptococcaceae, g.Peptococcus	0.002752	0.002229	0	0
Peptococcaceae, g.uncultured	0.004379	0.001625	0	0
Anaerovoracaceae, g.Family_XIII_UCG001	0.000717	0.000664	0	0
Akkermansiaceae, g.Akkermansia	1.88E-05	6.23E-05	0.06528	0.0648317

Supplementary Table T2 | Immune features assessed after antibiotic treatment in mice.

Technique	Tissue	Assessed parameters						
**Flow cytometry**	Spleen	T cells^						
	Placenta	T cells						
	MLN	T cells						
	ILN	T cells						
	Peritoneal cavity	T cells						
	Spleen	B cells^^						
	Placenta	B cells						
	Peritoneal cavity	B cells						
	Placenta	NK_CD3neg CD49bpos	CD11bpos F4/80pos	CD301pos				
**Luminex**	Splenocytes after 24h stimulation	IL-2	IL-6	IL-10	TNFa	IL-1b	IFNg	IL-22
	Amniotic fluid	IL-2	IL-6	IL-10	TNFa	IL-1b	IL-4	IL-5
**qPCR**	Colon	FOXP3	Gata3	RoryT	T-Bet			
	Placenta	FOXP3	Gata3	RoryT	T-Bet	IL-10		

^T cell panel: CD4pos, activated CD4pos, Th1 cells, Th2 cells, Th1Th2 ratio, Th17 cells, Treg cells, CD4pos, Neuropilin-1pos percent of Treg cells
^^ B cell panel: CD19pos, CD1dHi CD5pos, CD5pos CD1dpos, CD1dHi CD21Hi, B10, CD5neg, CD1dlow, CD5pos, CD1dlow, T2MZP, CD24neg CD21pos CD35pos, CD24pos CD21neg CD35neg, CD24pos CD21pos CD35pos, CD5neg CD23neg, CD5pos CD23neg, CD5pos CD23pos, CD1d histogram, CD5 histogram, CD21 histogram, CD23 histogram, CD24 histogram.
